# Benzene-1,2,4,5-tetra­carb­oxy­lic acid bis­(1,3,7-trimethyl-2,3,6,7-tetra­hydro-1*H*-purine-2,6-dione)

**DOI:** 10.1107/S1600536813022563

**Published:** 2013-08-17

**Authors:** Hadi D. Arman, Edward R. T. Tiekink

**Affiliations:** a Department of Chemistry, University of Texas at San Antonio, One UTSA Circle, San Antonio, TX 78249-0698, USA; b Department of Chemistry, University of Malaya, 50603 Kuala Lumpur, Malaysia

## Abstract

The asymmetric unit of the title co-crystal, C_10_H_6_O_8_·2C_8_H_10_N_4_O_2_, comprises a centrosymmetric benzene-1,2,4,5-tetra­carb­oxy­lic acid (*L*H_4_) mol­ecule and a mol­ecule of caffeine in a general position. *L*H_4_ is nonplanar, with the dihedral angles between the ring and pendent carb­oxy­lic acid groups being 44.22 (7) and 49.74 (7)°. By contrast, the caffeine mol­ecule is planar (r.m.s. deviation = 0.040 Å). Supra­molecular layers parallel to (-1-10) are sustained by carb­oxy­lic acid O—H⋯O(carbon­yl) and O—H⋯N(imidazole) hydrogen bonds, as well as by meth­yl–carbonyl C—H⋯O inter­actions. These stack *via* π–π inter­actions between the benzene and imidazole rings [inter-centroid distance = 3.4503 (10) Å].

## Related literature

For cocrystallization studies with benzene-1,2,4,5-tetra­carb­oxy­lic acid, see: Arman & Tiekink (2013 ▶).
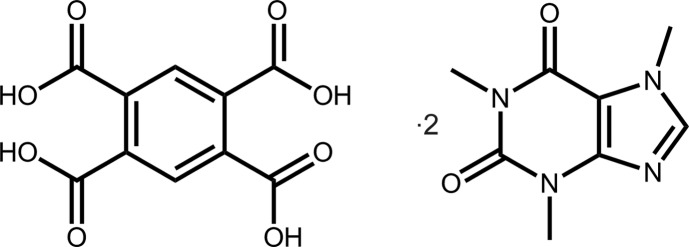


## Experimental

### 

#### Crystal data

C_10_H_6_O_8_·2C_8_H_10_N_4_O_2_
*M* *_r_* = 642.55
Triclinic, 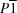

*a* = 7.4570 (15) Å
*b* = 9.0490 (15) Å
*c* = 11.782 (2) Å
α = 68.800 (11)°
β = 81.124 (13)°
γ = 73.441 (9)°
*V* = 709.3 (2) Å^3^
*Z* = 1
Mo *K*α radiation
μ = 0.12 mm^−1^
*T* = 98 K
0.55 × 0.30 × 0.25 mm

#### Data collection

Rigaku AFC12 Kappa/SATURN724 diffractometer
Absorption correction: multi-scan (*ABSCOR*; Higashi, 1995 ▶) *T*_min_ = 0.838, *T*_max_ = 1
4935 measured reflections
3213 independent reflections
3006 reflections with *I* > 2σ(*I*)
*R*_int_ = 0.020
Standard reflections: 0

#### Refinement

*R*[*F* ^2^ > 2σ(*F* ^2^)] = 0.044
*wR*(*F* ^2^) = 0.120
*S* = 1.04
3213 reflections
218 parameters
2 restraints
H atoms treated by a mixture of independent and constrained refinement
Δρ_max_ = 0.37 e Å^−3^
Δρ_min_ = −0.33 e Å^−3^

### 

Data collection: *CrystalClear* (Molecular Structure Corporation & Rigaku, 2005 ▶); cell refinement: *CrystalClear*; data reduction: *CrystalClear*; program(s) used to solve structure: *SHELXS97* (Sheldrick, 2008 ▶); program(s) used to refine structure: *SHELXL97* (Sheldrick, 2008 ▶); molecular graphics: *ORTEPII* (Johnson, 1976 ▶) and *DIAMOND* (Brandenburg, 2006 ▶); software used to prepare material for publication: *publCIF* (Westrip, 2010 ▶).

## Supplementary Material

Crystal structure: contains datablock(s) global, I. DOI: 10.1107/S1600536813022563/hg5340sup1.cif

Structure factors: contains datablock(s) I. DOI: 10.1107/S1600536813022563/hg5340Isup2.hkl

Supplementary material file. DOI: 10.1107/S1600536813022563/hg5340Isup3.cml

Additional supplementary materials:  crystallographic information; 3D view; checkCIF report

## Figures and Tables

**Table 1:** Hydrogen-bond geometry (Å, °)

*D*—H⋯*A*	*D*—H	H⋯*A*	*D*⋯*A*	*D*—H⋯*A*
O2—H1O⋯N3^i^	0.86 (1)	1.83 (1)	2.6774 (17)	171 (2)
O4—H2O⋯O5	0.84 (2)	1.84 (2)	2.6571 (15)	162 (2)
C12—H12*B*⋯O6^ii^	0.98	2.30	3.239 (2)	159

Symmetry codes: (i) 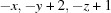; (ii) 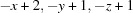.

**Fig. 1.**
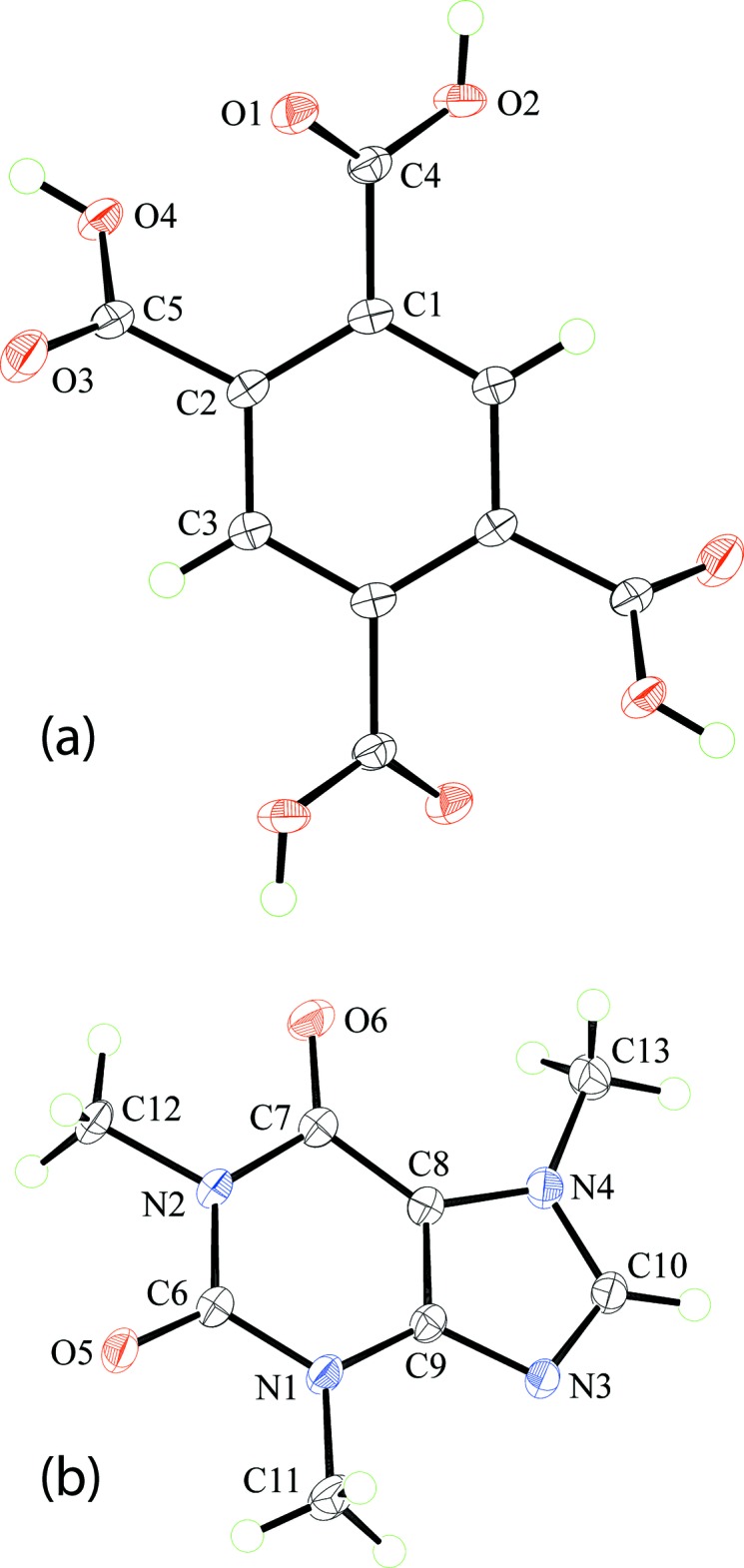
Molecular structures of the components of (I), showing atom-labelling scheme and displacement ellipsoids at the 50% probability level. The unlabelled atoms of HL4 (a) are generated by the symmetry operation 1 - x, 2 - y, 2 - z.

**Fig. 2.**
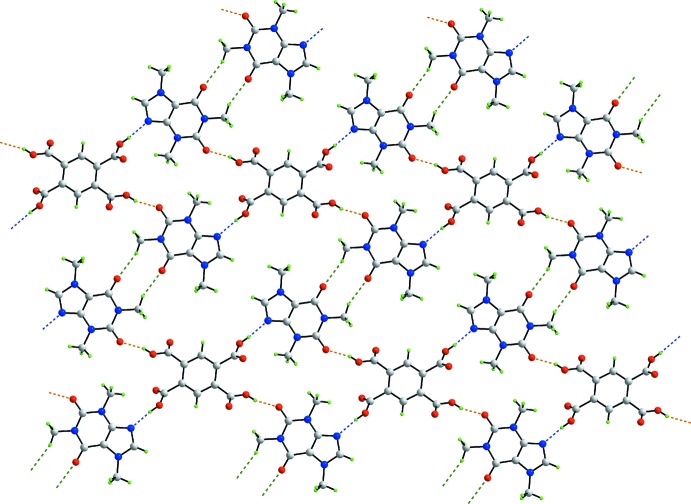
Views of the supramolecular layer in (I). The O—H···O (orange), O—H···N (blue) and C—H···O (green) interactions are shown as dashed lines.

**Fig. 3.**
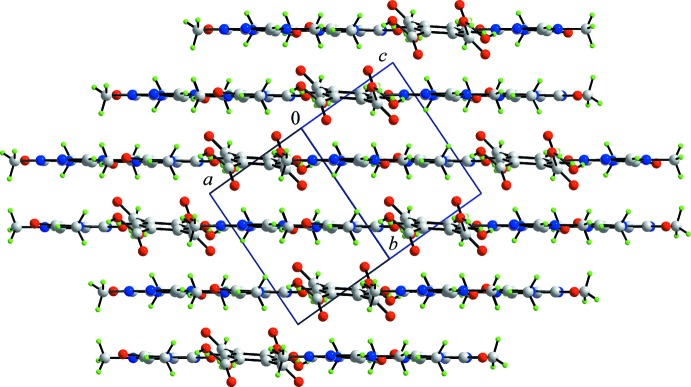
Unit-cell contents in (I) highlighting the stacking of layers shown in Fig. 2.

## supplementary crystallographic information

### 1. Comment

The title co-crystal was investigated in continuation of recent structural studies of the products obtained from the co-crystallization of benzene-1,2,4,5-tetracarboxylic acid (*L*H_4_) with various pyridyl-containing molecules (Arman & Tiekink, 2013).

The asymmetric unit comprises half a molecule of *L*H_4_, being disposed about a centre of inversion, and a molecule of caffeine in a general position, Fig. 1. Twists are evident in *L*H_4_ as seen in the dihedral angles of 44.22 (7) and 49.74 (7)° formed, respectively, between the O1- and O3-carboxylic acids and the benzene ring to which they are attached. The 14 non-hydrogen atoms of the caffeine molecule are co-planar with an r.m.s. deviation = 0.040 Å.

In the crystal packing, the H*L*_4_ molecule forms two O2—H···O5-carbonyl and two O4—H···N3-imidazole hydrogen bonds to form a supramolecular chain constructed about centrosymmetric 26-membered {···HO—C_4_OH···OCNCN}_2_ synthons, Table 1. Chains are connected into a layer approximately parallel to (110) by methyl-C12—H···O6(carbonyl) interactions *via* centrosymmetric and 10-membered {···OCNCH}_2_ synthons, Fig. 2. Layers are connected into a three-dimensional architecture by π–π interactions between the benzene and imidazolyl rings [inter-centroid distance = 3.4503 (10) Å, angle of inclination = 9.54 (7)° for symmetry operation *x*, *y*, 1 + *z*], Fig. 3.

### 2. Experimental

Crystals of (I) were obtained by the co-crystallization of caffeine (Sigma–Aldrich, 0.08 mmol) and benzene-1,2,4,5-tetracarboxylic acid (Sigma–Aldrich, 0.09 mmol) in acetone solution. Crystals were obtained by slow evaporation.

### 3. Refinement

C-bound H atoms were placed in calculated positions (C—H = 0.95–0.98 Å) and were included in the refinement in the riding-model approximation, with *U*_iso_(H) set to 1.2–1.5*U*_eq_(C). The O- and N-bound H atoms were located in a difference Fourier map and were refined with distance restraints of O—H = 0.84 (1) Å and N—H = 0.88 (1) Å, and with *U*_iso_(H) = 1.2*U*_eq_(N) and 1.5*U*_eq_(O).

### Crystal data

**Table:**

C_10_H_6_O_8_·2C_8_H_10_N_4_O_2_	*Z* = 1
*M**_r_* = 642.55	*F*(000) = 334
Triclinic, *P*1	*D*_x_ = 1.504 Mg m^−^^3^
Hall symbol: -P 1	Mo *K*α radiation, λ = 0.71073 Å
*a* = 7.4570 (15) Å	Cell parameters from 2842 reflections
*b* = 9.0490 (15) Å	θ = 3.3–40.4°
*c* = 11.782 (2) Å	µ = 0.12 mm^−^^1^
α = 68.800 (11)°	*T* = 98 K
β = 81.124 (13)°	Block, colourless
γ = 73.441 (9)°	0.55 × 0.30 × 0.25 mm
*V* = 709.3 (2) Å^3^	

### Data collection

**Table:**

Rigaku AFC12 Kappa/SATURN724 diffractometer	3213 independent reflections
Radiation source: fine-focus sealed tube	3006 reflections with *I* > 2σ(*I*)
Graphite monochromator	*R*_int_ = 0.020
ω scans	θ_max_ = 27.5°, θ_min_ = 1.9°
Absorption correction: multi-scan (*ABSCOR*; Higashi, 1995)	*h* = −9→9
*T*_min_ = 0.838, *T*_max_ = 1	*k* = −11→11
4935 measured reflections	*l* = −14→15

### Refinement

**Table:**

Refinement on *F*^2^	Primary atom site location: structure-invariant direct methods
Least-squares matrix: full	Secondary atom site location: difference Fourier map
*R*[*F*^2^ > 2σ(*F*^2^)] = 0.044	Hydrogen site location: inferred from neighbouring sites
*wR*(*F*^2^) = 0.120	H atoms treated by a mixture of independent and constrained refinement
*S* = 1.04	*w* = 1/[σ^2^(*F*_o_^2^) + (0.0679*P*)^2^ + 0.3025*P*] where *P* = (*F*_o_^2^ + 2*F*_c_^2^)/3
3213 reflections	(Δ/σ)_max_ < 0.001
218 parameters	Δρ_max_ = 0.37 e Å^−^^3^
2 restraints	Δρ_min_ = −0.33 e Å^−^^3^

### Special details

**Table:**

Geometry. All s.u.'s (except the s.u. in the dihedral angle between two l.s. planes) are estimated using the full covariance matrix. The cell s.u.'s are taken into account individually in the estimation of s.u.'s in distances, angles and torsion angles; correlations between s.u.'s in cell parameters are only used when they are defined by crystal symmetry. An approximate (isotropic) treatment of cell s.u.'s is used for estimating s.u.'s involving l.s. planes.
Refinement. Refinement of *F*^2^ against ALL reflections. The weighted *R*-factor *wR* and goodness of fit *S* are based on *F*^2^, conventional *R*-factors *R* are based on *F*, with *F* set to zero for negative *F*^2^. The threshold expression of *F*^2^ > σ(*F*^2^) is used only for calculating *R*-factors(gt) *etc*. and is not relevant to the choice of reflections for refinement. *R*-factors based on *F*^2^ are statistically about twice as large as those based on *F*, and *R*-factors based on ALL data will be even larger.

### Fractional atomic coordinates and isotropic or equivalent isotropic displacement parameters (Å^2^)

**Table:**

	*x*	*y*	*z*	*U*_iso_*/*U*_eq_	
O1	0.10476 (13)	0.94888 (12)	0.88177 (9)	0.0215 (2)	
O2	0.05750 (13)	1.21613 (12)	0.85531 (9)	0.0216 (2)	
H1O	−0.0516 (16)	1.226 (2)	0.8350 (17)	0.032*	
O3	0.56478 (16)	0.70162 (12)	0.84529 (9)	0.0283 (3)	
O4	0.43724 (14)	0.95115 (12)	0.71641 (8)	0.0202 (2)	
H2O	0.430 (3)	0.902 (2)	0.6695 (17)	0.051 (6)*	
O5	0.35494 (13)	0.85084 (12)	0.54930 (8)	0.0225 (2)	
O6	0.84545 (14)	0.50918 (14)	0.40808 (10)	0.0283 (2)	
N1	0.30711 (15)	0.79348 (14)	0.38575 (10)	0.0189 (2)	
N2	0.60054 (15)	0.68600 (13)	0.47529 (10)	0.0175 (2)	
N3	0.29341 (15)	0.71840 (13)	0.20867 (10)	0.0180 (2)	
N4	0.58825 (16)	0.56529 (14)	0.20875 (10)	0.0191 (2)	
C1	0.33603 (17)	1.03685 (15)	0.94094 (11)	0.0162 (3)	
C2	0.49837 (17)	0.92882 (15)	0.91321 (11)	0.0159 (2)	
C3	0.66122 (18)	0.89199 (15)	0.97280 (11)	0.0168 (3)	
H3	0.7709	0.8179	0.9544	0.020*	
C4	0.15397 (18)	1.06201 (16)	0.88843 (11)	0.0174 (3)	
C5	0.50294 (18)	0.84709 (16)	0.82237 (11)	0.0179 (3)	
C6	0.41768 (18)	0.78078 (16)	0.47369 (11)	0.0178 (3)	
C7	0.68657 (18)	0.59678 (16)	0.39564 (12)	0.0187 (3)	
C8	0.56313 (18)	0.62252 (15)	0.30563 (11)	0.0174 (3)	
C9	0.38190 (18)	0.71494 (15)	0.30277 (11)	0.0167 (3)	
C10	0.42496 (19)	0.62623 (16)	0.15447 (12)	0.0193 (3)	
H10	0.4046	0.6060	0.0844	0.023*	
C11	0.1111 (2)	0.8869 (2)	0.38356 (14)	0.0312 (3)	
H11A	0.0737	0.9370	0.2991	0.047*	
H11B	0.0969	0.9727	0.4189	0.047*	
H11C	0.0316	0.8135	0.4312	0.047*	
C12	0.71544 (19)	0.68114 (17)	0.56767 (12)	0.0217 (3)	
H12A	0.7238	0.7922	0.5549	0.033*	
H12B	0.8414	0.6118	0.5607	0.033*	
H12C	0.6579	0.6362	0.6491	0.033*	
C13	0.7597 (2)	0.46116 (18)	0.17183 (13)	0.0251 (3)	
H13A	0.7855	0.3524	0.2344	0.038*	
H13B	0.8650	0.5102	0.1623	0.038*	
H13C	0.7433	0.4514	0.0941	0.038*	

### Atomic displacement parameters (Å^2^)

**Table:**

	*U*^11^	*U*^22^	*U*^33^	*U*^12^	*U*^13^	*U*^23^
O1	0.0199 (5)	0.0252 (5)	0.0241 (5)	−0.0084 (4)	−0.0032 (4)	−0.0109 (4)
O2	0.0180 (5)	0.0209 (5)	0.0262 (5)	−0.0038 (4)	−0.0101 (4)	−0.0055 (4)
O3	0.0414 (6)	0.0210 (5)	0.0249 (5)	−0.0036 (4)	−0.0113 (4)	−0.0099 (4)
O4	0.0250 (5)	0.0225 (5)	0.0154 (4)	−0.0064 (4)	−0.0052 (3)	−0.0072 (4)
O5	0.0203 (5)	0.0306 (5)	0.0201 (5)	−0.0043 (4)	−0.0040 (4)	−0.0129 (4)
O6	0.0192 (5)	0.0350 (6)	0.0301 (5)	0.0028 (4)	−0.0086 (4)	−0.0147 (4)
N1	0.0148 (5)	0.0249 (6)	0.0188 (5)	−0.0036 (4)	−0.0036 (4)	−0.0093 (4)
N2	0.0159 (5)	0.0215 (5)	0.0163 (5)	−0.0054 (4)	−0.0039 (4)	−0.0059 (4)
N3	0.0194 (5)	0.0191 (5)	0.0172 (5)	−0.0051 (4)	−0.0042 (4)	−0.0065 (4)
N4	0.0196 (5)	0.0205 (5)	0.0193 (5)	−0.0056 (4)	−0.0017 (4)	−0.0083 (4)
C1	0.0166 (6)	0.0174 (6)	0.0148 (5)	−0.0062 (5)	−0.0038 (4)	−0.0030 (4)
C2	0.0186 (6)	0.0170 (5)	0.0140 (5)	−0.0067 (5)	−0.0030 (4)	−0.0048 (4)
C3	0.0173 (6)	0.0175 (5)	0.0163 (5)	−0.0050 (4)	−0.0029 (4)	−0.0049 (4)
C4	0.0167 (6)	0.0227 (6)	0.0139 (5)	−0.0064 (5)	−0.0019 (4)	−0.0060 (5)
C5	0.0184 (6)	0.0206 (6)	0.0174 (6)	−0.0061 (5)	−0.0036 (4)	−0.0075 (5)
C6	0.0175 (6)	0.0205 (6)	0.0163 (6)	−0.0067 (5)	−0.0023 (4)	−0.0050 (5)
C7	0.0180 (6)	0.0203 (6)	0.0180 (6)	−0.0056 (5)	−0.0022 (5)	−0.0057 (5)
C8	0.0178 (6)	0.0187 (6)	0.0161 (6)	−0.0052 (5)	−0.0015 (5)	−0.0056 (5)
C9	0.0163 (6)	0.0183 (6)	0.0164 (5)	−0.0064 (5)	−0.0022 (4)	−0.0046 (4)
C10	0.0228 (6)	0.0189 (6)	0.0176 (6)	−0.0064 (5)	−0.0042 (5)	−0.0058 (5)
C11	0.0177 (7)	0.0470 (9)	0.0312 (7)	0.0037 (6)	−0.0071 (5)	−0.0225 (7)
C12	0.0188 (6)	0.0291 (7)	0.0198 (6)	−0.0061 (5)	−0.0065 (5)	−0.0090 (5)
C13	0.0213 (6)	0.0279 (7)	0.0291 (7)	−0.0035 (5)	−0.0007 (5)	−0.0154 (6)

### Geometric parameters (Å, º)

**Table:**

O1—C4	1.2123 (16)	C1—C3^i^	1.3911 (17)
O2—C4	1.3176 (16)	C1—C2	1.4008 (18)
O2—H1O	0.855 (9)	C1—C4	1.5030 (17)
O3—C5	1.2057 (17)	C2—C3	1.3935 (17)
O4—C5	1.3270 (15)	C2—C5	1.4968 (17)
O4—H2O	0.843 (9)	C3—C1^i^	1.3911 (17)
O5—C6	1.2349 (16)	C3—H3	0.9500
O6—C7	1.2197 (17)	C7—C8	1.4236 (17)
N1—C9	1.3713 (17)	C8—C9	1.3698 (18)
N1—C6	1.3747 (16)	C10—H10	0.9500
N1—C11	1.4638 (17)	C11—H11A	0.9800
N2—C6	1.3875 (17)	C11—H11B	0.9800
N2—C7	1.4181 (17)	C11—H11C	0.9800
N2—C12	1.4671 (15)	C12—H12A	0.9800
N3—C10	1.3384 (17)	C12—H12B	0.9800
N3—C9	1.3618 (16)	C12—H12C	0.9800
N4—C10	1.3410 (17)	C13—H13A	0.9800
N4—C8	1.3829 (16)	C13—H13B	0.9800
N4—C13	1.4664 (17)	C13—H13C	0.9800
			
C4—O2—H1O	110.9 (13)	O6—C7—N2	121.91 (12)
C5—O4—H2O	111.5 (16)	O6—C7—C8	126.89 (13)
C9—N1—C6	119.13 (11)	N2—C7—C8	111.20 (11)
C9—N1—C11	121.00 (11)	C9—C8—N4	105.48 (11)
C6—N1—C11	119.85 (11)	C9—C8—C7	123.20 (12)
C6—N2—C7	126.45 (11)	N4—C8—C7	131.32 (12)
C6—N2—C12	116.05 (11)	N3—C9—C8	111.54 (11)
C7—N2—C12	117.50 (11)	N3—C9—N1	126.48 (12)
C10—N3—C9	103.47 (11)	C8—C9—N1	121.97 (11)
C10—N4—C8	106.08 (11)	N3—C10—N4	113.43 (11)
C10—N4—C13	127.01 (11)	N3—C10—H10	123.3
C8—N4—C13	126.90 (11)	N4—C10—H10	123.3
C3^i^—C1—C2	119.74 (12)	N1—C11—H11A	109.5
C3^i^—C1—C4	119.61 (11)	N1—C11—H11B	109.5
C2—C1—C4	120.31 (11)	H11A—C11—H11B	109.5
C3—C2—C1	119.94 (11)	N1—C11—H11C	109.5
C3—C2—C5	117.97 (11)	H11A—C11—H11C	109.5
C1—C2—C5	122.07 (11)	H11B—C11—H11C	109.5
C1^i^—C3—C2	120.31 (12)	N2—C12—H12A	109.5
C1^i^—C3—H3	119.8	N2—C12—H12B	109.5
C2—C3—H3	119.8	H12A—C12—H12B	109.5
O1—C4—O2	125.76 (12)	N2—C12—H12C	109.5
O1—C4—C1	121.79 (12)	H12A—C12—H12C	109.5
O2—C4—C1	112.42 (11)	H12B—C12—H12C	109.5
O3—C5—O4	125.01 (12)	N4—C13—H13A	109.5
O3—C5—C2	121.87 (11)	N4—C13—H13B	109.5
O4—C5—C2	113.10 (11)	H13A—C13—H13B	109.5
O5—C6—N1	120.74 (12)	N4—C13—H13C	109.5
O5—C6—N2	121.29 (11)	H13A—C13—H13C	109.5
N1—C6—N2	117.97 (11)	H13B—C13—H13C	109.5
			
C3^i^—C1—C2—C3	0.6 (2)	C6—N2—C7—C8	3.16 (18)
C4—C1—C2—C3	−172.74 (11)	C12—N2—C7—C8	−175.68 (10)
C3^i^—C1—C2—C5	179.23 (11)	C10—N4—C8—C9	0.39 (14)
C4—C1—C2—C5	5.92 (18)	C13—N4—C8—C9	179.52 (12)
C1—C2—C3—C1^i^	−0.6 (2)	C10—N4—C8—C7	179.29 (13)
C5—C2—C3—C1^i^	−179.28 (11)	C13—N4—C8—C7	−1.6 (2)
C3^i^—C1—C4—O1	−131.94 (13)	O6—C7—C8—C9	175.61 (13)
C2—C1—C4—O1	41.37 (18)	N2—C7—C8—C9	−3.34 (18)
C3^i^—C1—C4—O2	46.39 (15)	O6—C7—C8—N4	−3.1 (2)
C2—C1—C4—O2	−140.30 (12)	N2—C7—C8—N4	177.92 (12)
C3—C2—C5—O3	48.27 (18)	C10—N3—C9—C8	−0.07 (14)
C1—C2—C5—O3	−130.41 (14)	C10—N3—C9—N1	179.02 (12)
C3—C2—C5—O4	−130.22 (12)	N4—C8—C9—N3	−0.20 (14)
C1—C2—C5—O4	51.09 (16)	C7—C8—C9—N3	−179.22 (11)
C9—N1—C6—O5	179.55 (11)	N4—C8—C9—N1	−179.34 (11)
C11—N1—C6—O5	−1.85 (19)	C7—C8—C9—N1	1.6 (2)
C9—N1—C6—N2	−1.00 (18)	C6—N1—C9—N3	−178.25 (12)
C11—N1—C6—N2	177.60 (12)	C11—N1—C9—N3	3.2 (2)
C7—N2—C6—O5	178.31 (12)	C6—N1—C9—C8	0.75 (19)
C12—N2—C6—O5	−2.83 (18)	C11—N1—C9—C8	−177.83 (13)
C7—N2—C6—N1	−1.14 (19)	C9—N3—C10—N4	0.33 (15)
C12—N2—C6—N1	177.72 (11)	C8—N4—C10—N3	−0.47 (15)
C6—N2—C7—O6	−175.85 (12)	C13—N4—C10—N3	−179.60 (12)
C12—N2—C7—O6	5.31 (19)		

Symmetry code: (i) −*x*+1, −*y*+2, −*z*+2.

### Hydrogen-bond geometry (Å, º)

**Table:**

*D*—H···*A*	*D*—H	H···*A*	*D*···*A*	*D*—H···*A*
O2—H1*O*···N3^ii^	0.86 (1)	1.83 (1)	2.6774 (17)	171 (2)
O4—H2*O*···O5	0.84 (2)	1.84 (2)	2.6571 (15)	162 (2)
C12—H12*B*···O6^iii^	0.98	2.30	3.239 (2)	159

Symmetry codes: (ii) −*x*, −*y*+2, −*z*+1; (iii) −*x*+2, −*y*+1, −*z*+1.
